# Effect of internet‐based attention bias modification on the anxiety of Japanese workers: A randomized controlled trial

**DOI:** 10.1002/1348-9585.12229

**Published:** 2021-05-01

**Authors:** Jun Tayama, Akihito Shimazu, Sayaka Ogawa, Naoki Nakaya

**Affiliations:** ^1^ Faculty of Human Sciences Waseda University Saitama Japan; ^2^ Faculty of Policy Management Keio University Kanagawa Japan; ^3^ Center for Health and Community Medicine Nagasaki University Nagasaki Japan; ^4^ Department of Health Sciences Saitama Prefectural University Saitama Japan

**Keywords:** anxiety, attention bias modification, internet, Japanese, randomized controlled trial, worker

## Abstract

**Objectives:**

This study comprised a randomized controlled trial to examine the effect of internet‐based attention bias modification (iABM) on reducing workers' anxiety.

**Methods:**

In total, 300 eligible participants were randomized according to sex and age; 180 were assigned to the intervention group and 120 to the control group. The word stimuli used in the iABM included eight positive words and eight neutral words. Participants were instructed to indicate the positive word's position as quickly and accurately as possible by tapping one of the two directions on display. The intervention included five sessions per participant over 1 month, resulting in a total of 600 trials. The main outcome measure was the total state anxiety score of the State‐Trait Anxiety Inventory (STAI).

**Results:**

There was no significant difference in the net change in STAI scores between the intervention and control groups. The mean reaction time of the fifth session was significantly shorter than the mean reaction time of the first session. Furthermore, although there was no effect on the index of effect size Δ, the paired *t*‐test showed a significant decrease in the anxiety score.

**Conclusions:**

The iABM intervention in this study did not enhance the amelioration of workers' anxiety when compared with the control condition.

## 1 INTRODUCTION

Workers can experience serious occupational problems.^1^, ^2^, ^3^ In particular, occupational stress, such as overwork,^4^ is a problem that can lead to overwork‐related death.^5^ Occupational stress can affect both physical and mental health. For example, occupational stress caused by long working hours is associated with heart disease,^6^, ^7^ cerebrovascular disease,^7^ diabetes,^8^ and other risk factors. Furthermore, studies have shown that occupational stress and long working hours are associated with depression.^9^, ^10^ Anxiety is also related to long working hours—a feature of occupational stress.^9^, ^11^, ^12^ A meta‐analysis of the relationship between job satisfaction and health showed that job satisfaction was strongly associated with psychological problems, such as depression and anxiety.^13^ Also, individuals exposed to a high level of psychological job stress are approximately twice as likely to develop generalized anxiety disorder than those with a low level of job demands.^14^ Conversely, if occupational satisfaction is high, workers' anxiety tends to be low.^15^ High anxiety is also associated with high medical costs and absenteeism.^16^

The state anxiety of Japanese workers is higher for males than for females and for younger people than for middle‐aged people.^17^ A worker's high level of anxiety is affected by various work‐related factors, and the anxiety itself can be an obstacle to performing the work.^18^, ^19^, ^20^, ^21^ Psychosocial factors related to the working environment, such as job demands, poor job management, lack of compensation, and low social support, have been found to increase workers' anxiety.^18^ Further, a high level of anxiety has been shown to reduce productivity at work.^19^, ^20^, ^21^ In developed countries, the anxiety of general workers is likely to rise, causing a reduction in productivity in the workplace, which may be causing a variety of problems to others.^22^ Anxiety has potentially detrimental consequences for employees and organizations in the form of reduced job performance.^22^ It was revealed in a three‐wave study of 770 police officers that workplace anxiety negatively impacted job performance through cognitive interference and emotional exhaustion.^23^ Workplace anxiety affects job performance, since high levels of anxiety interfere with the ability to perform tasks, resulting in reduced levels of performance.^24^ Given that job performance is important to the organization and its employees, it seems very important to have a strategy to reduce anxiety in general workers. Thus, workers' anxiety is a mental health problem that should be clinically targeted.

Recently, attention bias modification (ABM) has become popular as a method for self‐managing psychological abnormalities.^25^, ^26^ Attention bias is a specific bias that a person with anxiety exhibits during information processing in which he/she tends to pay more attention to negative stimuli.^27^ When this tendency is strong, it may result in psychological abnormalities. A computer program that measures attention bias^25^ and implements ABM has been developed as a new method for normalizing psychological abnormalities. ABM is a computer‐based training method with various methods of trial. For example, ABM promotes cognitive processing for neutral stimulation rather than negative stimulation through repeated trials of simultaneous presentation of negative and neutral stimuli. By performing ABM, the capture time for neutral stimulation is shortened, and when attention bias is corrected, the anxiety of individuals with high anxiety levels is alleviated.^5^ Randomized controlled trials of participants with anxiety disorders have demonstrated that ABM can significantly attenuate anxiety symptoms compared to a non‐ABM group.^28^, ^29^ Similarly, a systematic review of ABM has corroborated its ability to reduce patients' anxiety.^30^

The ABM self‐management method is also available on the internet.^29^, ^31^, ^32^ In recent years, the internet has provided a new channel for psychological treatment. Internet‐based interventions are easily accessible, have a low cost, and can address different types of disorders.^33^ Moreover, as the assessments and interventions are conducted online, clients can avoid the fear of meeting others, such as in face‐to‐face sessions with clinicians.^34^ In addition, many randomized controlled trials have shown that self‐managed internet‐based cognitive‐behavioral therapy (iCBT) is effective for reducing anxiety.^33^, ^35^, ^36^ Thus, internet‐based attention bias modification (iABM), which is similar to iCBT, may also be an effective self‐management method for reducing anxiety.

However, to date, no study has examined the anxiety‐reducing effect of iABM in a large sample of workers. In other words, it is unclear whether iABM contributes to reducing workers' anxiety. In terms of mental health issues in the industrial and labor fields, iABM is insufficiently verified regarding its implementation among workers.

Therefore, this study comprised a randomized controlled trial with workers to examine the effects of iABM. The purpose of this study was to assess whether iABM is an effective strategy for reducing workers' anxiety. The main and secondary hypotheses of this study are as follows.

1. Workers' state anxiety is normalized by implementing iABM.
2. Workers' state anxiety is normalized more in men than in women and more in younger people than in middle ages by implementing iABM.

## 2 METHODS

### 2.1 Study design

This study was a randomized controlled trial that involved two groups: an iABM group that received the iABM intervention and a control group that did not receive the intervention. The iABM group received 1 month of iABM.

### 2.2 Survey procedure

Participants were recruited from an online panel database provided by a Japanese research company (Macromill, Inc, Tokyo, Japan). Participants, with an equal sex and age distribution, were randomly assigned to the iABM and control groups.

Participants were informed of the study's aim and the intended use of the survey data and were guaranteed anonymity. Individuals who agreed to the stated procedures and conditions were permitted to participate and completed demographic questions via the internet. Each participant received approximately US$10 for their participation through the Macromill, Inc system. Those who participated in the intervention as well received an additional US$10. In the intervention group, anxiety, which is the primary endpoint, was measured at the baseline and at the end of the intervention. Similarly, measurements matching the intervention group were performed at two points for the non‐intervention group. Since the individuals' data were acquired through an internet research company, it is not appropriate for public deposition. However, in terms of data availability, all relevant data are analyzed in this article.

### 2.3 Participants

The recruitment criteria were that workers should be aged 20‐59 years and employed full‐time in Japan. The exclusion criteria included mental illness, heart disease, company managers, company officers, and foreign workers. The 300 people who were eligible to participate were randomized according to sex and age and assigned to the intervention group (180 people) or control group (120 people). The mean age ± SD was 39.8 ± 10.3 years in the intervention group and 39.6 ± 10.3 years in the control group (*P* = .93).

### 2.4 Attention training task

The word stimuli used in the iABM included eight positive words and eight neutral words. In this study, two Japanese kanji characters were used as word stimuli in all trials to control the time required for visual information processing. In each trial, randomized pairs of neutral and positive words were displayed against a white background in the upper and lower portions of the computer screen. The word presentation was as follows: a fixation cross was displayed for 500 ms, followed by the presentation of the pair of target words for 500 ms, and then a symbol (“:”) was displayed at the bottom of the screen until a button press response was registered. Participants were instructed to indicate the positive word's position as quickly and accurately as possible by tapping one of the two directions on the display. Each session included 120 trials. The intervention included five sessions per participant over a 1‐month period, resulting in a total of 600 trials. Previous studies have shown that even with a relatively small total number of trials (600 trials), ABM is effective in reducing anxiety.^30^, ^37^ The reaction time of attention bias was measured during iABM.

### 2.5 Outcomes

The main outcome measure was the total state anxiety score of the State‐Trait Anxiety Inventory (STAI)^38^ at 4 weeks after the treatment had begun.

### 2.6 Sample size

The sample size was calculated based on a previous study (n = 29) of ABM that used the state anxiety subscale of the STAI (STAI‐S) as the outcome measure.^39^ The study found a reduction in the mean STAI‐S score after 4 weeks of the intervention (mean ± SD STAI‐S score at baseline: 59.1 ± 10.5; at 4 weeks: 51.1 ± 9.8). From this, it was estimated that ≥36 cases per group were required for a difference in the STAI‐S score ≥8.0 (SD = 10.0), with an α level of 0.05 (two‐tailed) and 80% power.

### 2.7 Statistical analysis

All analyses were conducted according to the intention‐to‐treat principle. The regression equation *y* = *ax* + b was used as a method of complementing the data loss, in which the STAI value of the baseline was *x* and the STAI value after the intervention was *y*. Analysis of covariance (ANCOVA) was used to assess the mean STAI scores' differences, and 95% confidence intervals (CIs) and *P*‐values were calculated. The covariates in the ANCOVA were sex, age, and the STAI scores at baseline. A two‐tailed test was used with the α level set at 0.05%. The Bonferroni correction was used for the secondary analysis, which estimated that the differences were significant if *P* < .017 (0.05 ÷ 3). SPSS version 26.0 (IBM Corp., Armonk, NY) was used for all statistical analyses.

## 3 RESULTS

### 3.1 Demographic data

**FIGURE 1:**
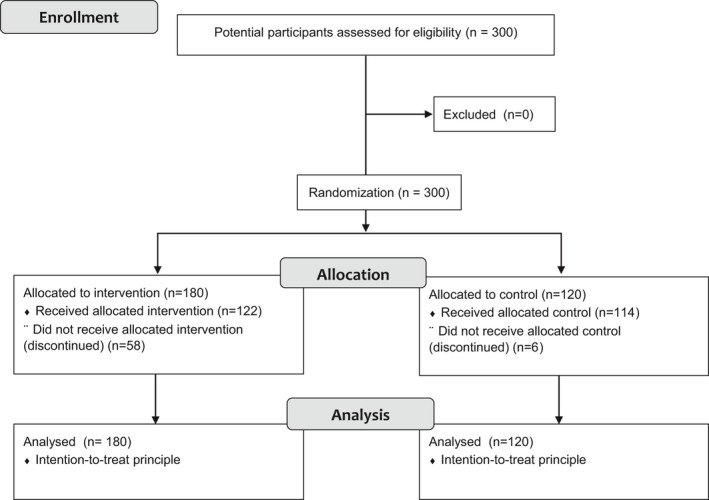
Recruitment, eligibility, and stratification of the participants in the study

A total of 300 potential participants were assessed for eligibility, and written informed consent was obtained. The iABM intervention and control groups were randomly assigned 180 and 120 participants, respectively. The 1‐month study was completed by 236 participants (79%) (Figure 1).

Table 1 shows baseline demographic data and reference values from a previous study.^17^ No significant differences were observed between the two groups.

**TABLE 1:** Baseline characteristics of the participants

Characteristics	Control, n = 120	iABM, n = 180	*P*‐value	Reference value (Japanese worker) (35)
Age, mean (SD), years	39.6 (10.3)	39.8 (10.3)	.92	Males: 39.3 (10.7), Females: 31.5 (9.7)
Sex, n (%)				
Male	60 (50)	90 (50)	1.00	1509
Female	60 (50)	90 (50)		353
Baseline state anxiety, mean (SD)				
STAI	45.4 (11.9)	45.4 (11.0)	.57	Males: 43.9 (9.2), Females: 43.1 (9.1)

Abbreviations: iABM, internet‐based attention bias modification; SD, standard deviation; STAI, State‐Trait Anxiety Inventory.

### 3.2 The average reaction time of attention bias

The average reaction time for iABM in this study ± SD is as follows: first session, 880 ± 103 ms, and in the fifth session, 785 ± 103 ms; a significant reduction in reaction time for all participants (*t* = 15.9, *P* < .001, *df* = 299).

### 3.3 Primary outcome measure

**FIGURE 2:**
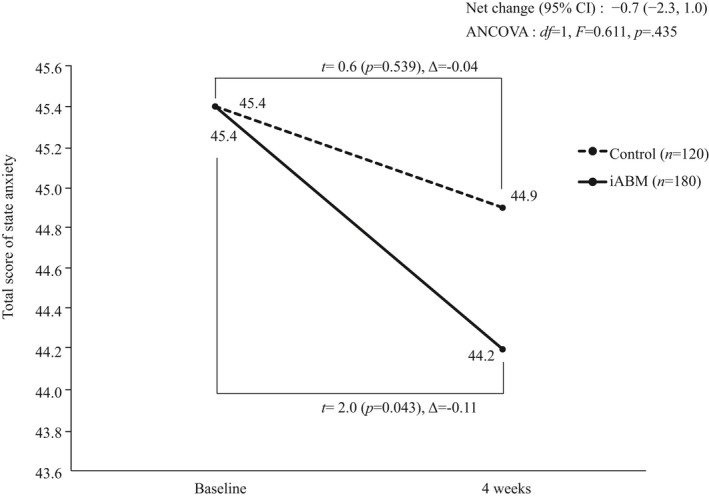
Main analysis of the state anxiety score of the State‐Trait Anxiety Inventory at baseline and 4 weeks. STAI, State‐Trait Anxiety Inventory; CI, confidence interval; ANCOVA, analysis of covariance; *df*, degrees of freedom; iABM, internet‐based attention bias modification. The ANCOVA was adjusted for age (continuous variables), sex, and baseline state anxiety score (continuous variables). The *P*‐value was calculated using the Bonferroni correction. The data were deemed significant in the main analyses if *P* < .05 and <0.01666

Figure 2 summarizes the data of the iABM group following treatment and the control group after 4 weeks. The primary index was the STAI score. No significant difference in the net change in the STAI score between the intervention and control groups (−0.7; 95% CI, −2.3 to 1.0; *P* = .44) was found; however, the intervention group had significantly lower STAI scores after 4 weeks of treatment compared with their baseline scores (control: *t* = 0.6, *P* = .54; iABM: *t* = 2.0, *P* = .043).

A secondary analysis was conducted by dividing the groups by sex, age (≤39 or ≥40 years), and baseline STAI score (male state anxiety score ≤40 or ≥41, and female state anxiety score ≤41 or ≥42). However, no significant difference in the net change in the STAI score for any factor was found (Table 2).

**TABLE 2:** Secondary analysis of the state anxiety score of the State‐Trait Anxiety Inventory at baseline and 4 weeks

Group	n	Baseline, mean (SD)	4 Weeks, mean (SD)	Paired *t*‐test		Net change (95% CI)	ANCOVA
				*t*	*P*‐value		*P*‐value*
Male							
Control	60	45.6 (13.2)	44.6 (10.4)	0.9	.394	0.1 (−1.8 to 2.1)	.888
iABM	90	46.4 (10.8)	45.3 (9.7)	1.8	.077		
Female							
Control	60	45.2 (10.6)	45.2 (10.8)	−0.1	.991	−1.3 (−3.8 to 1.3)	.327
iABM	90	44.4 (11.1)	43.2 (10.7)	1.2	.219		
Age, ≤39							
Control	60	45.5 (11.9)	46.1 (9.9)	−0.5	.634	−1.0 (−3.5 to 1.5)	.420
iABM	90	44.9 (11.1)	44.7 (9.7)	0.2	.874		
Age, ≥40							
Control	60	45.2 (12.0)	43.7 (11.1)	1.6	.115	−0.5 (−2.6 to 1.6)	.660
iABM	90	45.9 (10.9)	43.7 (10.8)	3.2	.002		
Male, and state anxiety ≤40							
Control	21	32.5 (6.2)	36.3 (1.4)	−2.4	.025	−1.2 (−4.7 to 2.2)	.469
iABM	23	32.9 (5.2)	35.2 (5.1)	−2.3	.033		
Male, and state anxiety ≥41							
Control	39	52.6 (10.2)	49.1 (9.4)	2.6	.013	1.2 (−1.6 to 4.0)	.404
iABM	67	51.1 (7.9)	48.7 (8.4)	3.1	.003		
Female, and state anxiety ≤41							
Control	20	33.9 (4.9)	36.9 (7.4)	−1.7	.114	0.6 (−4.0 to 5.3)	.784
iABM	35	33.5 (5.0)	36.6 (7.5)	−2.2	.036		
Female, and state anxiety ≥42							
Control	40	50.8 (7.7)	49.3 (9.9)	1.2	.255	−2.5 (−5.9 to 0.9)	.146
iABM	55	51.3 (7.9)	47.4 (10.3)	3.4	.001		

Note:

The ANCOVA was adjusted for age (continuous variables), sex, and baseline state anxiety score (continuous variables).

Abbreviations: ANCOVA, analysis of covariance; CI, confidence interval.

* The *P*‐value was calculated using the Bonferroni correction. The data were deemed significant in the main and secondary analyses if *P* < .05 and *P* < .01666, respectively.

## 4 DISCUSSION

In this study, a randomized controlled trial was conducted to examine the effect of iABM on reducing workers' anxiety. No statistical difference in efficacy between the iABM and control groups was observed. The secondary analyses that considered the factors of sex, age, and differences in the baseline STAI score did not alter these results. Therefore, our hypothesis that iABM would enhance the improvement in workers' anxiety was not supported. Similarly, the hypothesis that workers' state anxiety is normalized more in men than in women and more in younger people than in middle ages by implementing iABM was not supported.

One reason for the lack of benefit from the iABM may be related to the participants' degree of anxiety in this study and those in previous studies. The participants in previous studies had a considerably high level of anxiety, and the mean ± SD of the state anxiety indexed by the baseline STAI was 59.1 ± 10.5.^39^, ^40^ In contrast, participants' anxiety in this study was not very high, and the average ± SD was 45.4 ± 11.0. A meta‐analysis of ABM has shown that it is more effective for people with a high anxiety level than for healthy people.^30^

Another reason for the lack of advantage may be that the ABM was provided online rather than in a medical setting. Previous randomized controlled trials have shown that ABM targeting social anxiety disorders in the actual field of medical care can contribute to anxiety reduction.^39^, ^41^ Alternatively, although a randomized, double‐blind placebo‐controlled trial investigating the anxiety‐reducing effect of iABM for social anxiety disorder found a significant main effect of time, no statistically significant interaction was found for efficacy between the iABM and placebo groups.^29^ The effect size of the ABM group in a randomized controlled trial of ABM that was conducted face‐to‐face was delta = −1.33 (a large effect size),^39^ whereas the effect size of the iABM group in this study was delta = −0.11 (no effect). The expected anxiety‐relieving effect was possibly not obtained because participants were more relaxed without the presence of health‐care workers during the ABM implementation.^42^

However, repeated iABM training showed a significant reduction in reaction time. It has long been known that reaction time and anxiety level in the experimental attention bias paradigm are correlated.^27^ The mean reaction time of the fifth session was significantly shorter than the mean reaction time of the first session. Furthermore, although there was no effect on the index of effect size Δ, the paired *t*‐test showed a significant decrease in the anxiety score. These findings suggest that the intervention element of iABM normalizes attention bias but does not sufficiently reduce anxiety. In addition, combination interventions that add other intervention elements, such as e‐learning to iABM,^32^, ^43^ may reduce anxiety.

An advantage of this study is that it was a randomized controlled trial that was able to control known and unknown confounding factors. The disadvantage of this study is that fewer people completed the intervention than in previous studies.

### 4.1 Limitations

This study has some limitations. First, many participants dropped out during the study. Only 236 (78.6%) of the 300 participants completed the 4‐week study. There were no dropouts in a previous study used as the reference for estimating the sample size.^40^

The second limitation is related to the number of sessions and the total number of iABM intervention trials. This study's 4‐week intervention period was the same length as the previous study^40^ that was referred to for the sample size estimation. However, the total number of sessions during the intervention period was five in this study and eight in previous studies. Furthermore, when comparing the total number of trials, there were 600 in this study and 1920 in previous studies, which is more than three times as many as this study. The small number of sessions and the low total number of trials may have affected the results.

The third limitation is that the socioeconomic status of participants may have affected the results. The participants are considered to have a relatively high socioeconomic status, given their access to the internet. High socioeconomic status is associated with a low prevalence of mental health problems and a high level of social support.^44^ It is assumed that it is easy for this demographic to access resources to help them resolve their anxiety, obtain solutions to problems, and access information about self‐management. In other words, the high socioeconomic status of participants may have reduced the difference between the intervention group and the control group in this study.

A fourth limitation is that the scale used to measure anxiety may have not been appropriate. Since occupational stress was not investigated in this study, future studies could focus on work‐related anxiety.

A fifth limitation is that the participants are internet‐savvy and may have a high educational level. Therefore, the generalizability of this study's findings is unclear.

## 5 CONCLUSION

In conclusion, while iABM is effective for improving symptoms of anxiety, the iABM intervention in this study did not enhance the amelioration of anxiety when compared with the control condition. In the future, it will be important to obtain further evidence from randomized controlled trials and to examine the relationship between iABM and anxiety. The continuous pursuit of additional effective intervention methods will likely reduce workers' anxiety and further improve their productivity.

## CONFLICTS OF INTEREST

Dr Tayama (JT) received support in the form of a grant from the Japan Productivity Center in Tokyo, Japan. The remaining authors received no financial support or funding for this manuscript. The funding source had no role in the design, practice, or analysis of this study.

## DISCLOSURE

*Approval of the research protocol:* The Ethics Committee of Nagasaki University (no. 18072701) approved this study's protocol. This study was conducted according to the 1964 Declaration of Helsinki's ethical standards and its later amendments. *Informed consent:* Written informed consent was obtained from all individual participants included in the study. *Registry and the registration no*.: This study was registered at https://upload.umin.ac.jp/cgi‐open‐bin/ctr/ctr_view.cgi?recptno=R000046962 (UMIN Clinical Trials Registry, UMIN000041266). *Animal studies:* N/A.

## AUTHOR CONTRIBUTIONS

JT and AS designed this study. JT organized data collection. JT, SO, and NN conducted statistical analysis. JT and NN prepared for original draft. AS and SO reviewed and edited the draft. All authors have read and approved the final manuscript's submission for publication.
